# Breast adenoid cystic carcinoma in a 19-year-old man: a case report and review of the literature

**DOI:** 10.1186/s12957-015-0442-8

**Published:** 2015-02-06

**Authors:** Peng Tang, Shiping Yang, Xiaojie Zhong, Jia Yao, Yu Zhang, Huaying Dong, Guanqiao Li

**Affiliations:** Department of Breast surgery, Hainan Province People’s Hospital, NO.19, Xiuhua Road, Xiuying District, Haikou, Hainan 570311 China; Department of Radiation Oncology, Hainan Province People’s Hospital, NO.19, Xiuhua Road, Xiuying District, Haikou, Hainan 570311 China

**Keywords:** Male, Breast, Adenoid cystic carcinoma, Treatment, Prognosis

## Abstract

Adenoid cystic carcinoma (ACC) of the breast is very rare in males. There have been only eight previous articles published on breast ACC in males. Here, we will report on the first case of this type of tumor in the Chinese province of Hainan. The patient was a 19-year-old male, and he underwent a radical mastectomy (RM) with axillary lymph node dissection. The histopathological examination specimen revealed that surgical margins were negative; none of the 41 axillary lymph nodes excised were positive for malignancy. The patient is alive and well 67 months after radical mastectomy. In the present study, we discuss the diagnosis and treatment options for male breast ACC based on previous English publications.

**Electronic supplementary material:**

The online version of this article (doi:10.1186/s12957-015-0442-8) contains supplementary material, which is available to authorized users.

## Background

Breast adenoid cystic carcinoma (ACC), previously called “cylindroma,” was first described by Geschickter and Copeland [1]. It is a rare neoplasm accounting for less than 0.1% of all patients diagnosed with breast cancer [2]. The uncommon gender (male) and rare subtype (adenoid cystic carcinoma) of the disease make “male breast adenoid cystic carcinoma” exceedingly rare.

The optimal management for male breast adenoid cystic carcinoma is unclear. Guidelines for diagnosis and treatment are based on those used for female breast ACC.

We have reviewed all the cases which occurred in our hospital from January 1994 to January 2014 and there was only one case of male breast ACC in all that time. This is the case of the 19-year-old patient that was mentioned earlier.

To our knowledge, only eight previous articles deal with breast ACC in males [3-10]. We will report the first case in Hainan Province of this unusual tumor and its status after 67 months of follow-up. The objective of this article is to draw attention to the diagnosis and to the treatment options for this tumor type.

## Case presentation

The patient is a 19-year-old man. The painless palpable mass in the right chest wall was approximately 20 mm in diameter, and he reported that the mass had persisted for the past 4 years. The patient qualified for a biopsy in a local county-level hospital. The pathologist then confirmed an adenoid cystic carcinoma. The specimen examination showed a mixed tubular and cribriform pattern. The estrogen receptor (ER) was positive at 3%. The sample was negative for progesterone receptor (PR) and c-erbB2. Then, he came to the thoracic surgery department of our hospital in February 2009. Physical examination revealed a 20 mm × 21 mm size subareolar mass which was irregularly shaped without fixation to the skin or muscle. There was no clinical evidence of regional lymphadenopathy. The patient had no past medical history or family history of cancer, and the patient did not smoke or drink alcohol.

**Figure 1:**
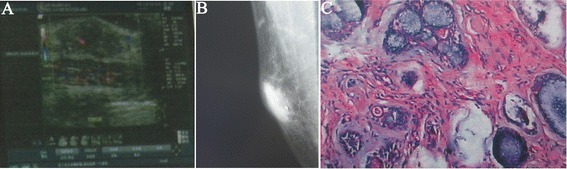
**Ultrasonography, mammography, and histopathological examination of male breast adenoid cystic carcinoma. (A)** Ultrasonography revealed an irregular, mixed echoic, partial compressible mass in the subareolar region. **(B)** Mammography (mediolateral oblique) revealed a spiculate hyperdense lesion. **(C)** Microscopic picture of male ACC stained by H&E stain (original magnification × 50) shows a predominant cribriform pattern and is composed of pseudocysts, filled with basophilic mucin, surrounded by basaloid cells. Neoplastic cells were arranged in cribriform and tubular.

A breast ultrasound and mammography were performed. The ultrasonography (USG) revealed an irregular, mixed echoic, partial compressible mass(21 mm × 20 mm × 9 mm) in the subareolar region of the right breast (Figure 1A). Mammography (Figure 1B) revealed a spiculate hyperdense lesion (30 mm × 15 mm) in the right breast in accordance with the findings of the ultrasonography. Serum tumor markers and other routine blood tests were normal. The chest X-ray, bone scan, axillary lymph node, and abdominal ultrasound were normal. Following on from the diagnosis, he underwent a radical mastectomy (RM) with axillary lymph node dissection. The pathological examination of the resection specimen revealed a 30 mm × 20 mm × 15 mm tumor. The histopathological examination specimen revealed (Figure 1C) that surgical margins were negative; none of the 41 axillary lymph nodes excised were positive for malignancy. Microscopic examination revealed that the tumor is composed of pseudocysts, filled with basophilic mucin, surrounded by basaloid cells. Neoplastic basaloid cells were arranged in cribriform and tubular. The neoplastic cells were negative for estrogen receptor expression, progesterone receptor expression, and HER2/neu staining by immunohistochemistry. The tumor was pathologically staged as pT2N0M0. Since the estrogen receptor was 3% positive in the biopsy, it was decided for the patient to be treated with tamoxifen at a daily dose of 20 mg for 5 years. But tamoxifen was terminated after 13 months because of sexual dysfunction. Neither chemotherapy nor radiotherapy was performed in the present case. After 67 months of post-operative follow-up, our patient remains free of loco-regional recurrence or distant metastases.

## Discussion

According to previous reports, ACC of the breast occurs predominantly in older female patients, with a median age range of 58–64 years [11]. The age distribution in males is not clear due to the lack of data about this subject. However, according to these eight cases of male breast ACC that have been documented [3-10], the median age at diagnosis was 37 years (range 13–82 years); four (50%) patients were diagnosed between the ages of 10 and 29. ACC of the breast occurs predominantly in younger male patients which is different from female patients.

Male breast cancer most frequently presents as a firm, painless breast mass, along with palpable axillary nodes, nipple retraction, and ulceration [12]. The mass is usually located in the subareolar region, but can also be seen in the upper outer quadrant [13]. Although ACC of the breast is also present as a subareolar mass and is generally multifocal [14], nipple discharge is not a common presenting symptom. ACC of the breast is rarely bilateral and has no tendency for a particular side of the breast [15]. The published tumor sizes of male breast ACC ranges from 1.8 to 3.7 cm [11]. The patient in the present study complained of a subareolar mass (2 cm) in the upper outer quadrant.

ACC of the breast shows pathological findings similar to ACC of the salivary gland. ACC has three varied growth patterns: glandular, tubular, and solid. Ro [16] classified ACC into three grades of tumor on the basis of the solid component as grade 1, completely glandular and cystic; grade 2, <30% solid component; and grade 3, >30% of solid components. All grade 3 tumors appear to behave like high-grade ductal breast cancer.

ACC of the breast in females usually has very favorable biological characteristics for treatment [2]. ACC of the breast belongs to the basal-like subgroup of breast cancers [17]. Based on extensive molecular and genetic profiling studies, basal-like tumors are most often hormone receptor (ER and PR) negative and do not express human epidermal growth factor receptor 2 (HER2) [18,19]. Unlike other triple-negative breast cancers that are associated with poor prognosis, breast ACC has an overall excellent prognosis [2]. The incidents of axillary lymph node metastases and distant metastases are lower [20]. It is because of these distinct clinic-pathologic features that set it apart from the other triple-negative breast cancers [11]. In the present case, immunohistochemistry (IHC) of post-operation was negative for ER, PR, and HER2.

Guideline for diagnosis and treatment in male breast ACC is not clear due to the extremely low levels of cases. Surgery is a mainstay of treatment for breast ACC. The various options for treatment include lumpectomy, wide excision with or without radical radiation, or modified radical mastectomy. Axillary lymph node dissection is rarely required because of the low incidence of spread to the axillary lymph nodes. Study in ACC of the breast shows that good local control can be achieved by lumpectomy with radiation or by simple mastectomy. Axillary lymph node dissection is worthless considering the rarity of lymph node involvement [21]. In the present case, a great number (41) of axillary lymph nodes were dissected, and none of the 41 axillary lymph nodes excised were positive for malignancy. This is very different from previous reports. In the past, radical mastectomy was preferred, and the rationale for this was the localized lesion being close to the pectoralis major muscle and the tumor being in a more advanced stage in men compared to that in women at the time of diagnosis [22]. In the present days, less extensive surgery is preferred such as modified radical mastectomy (MRM), which is an ontologically equivalent RM [23]. In 2009, there is no independent department of breast surgery in our hospital. The doctors in the thoracic surgery department performed the breast cancer operation, but they also performed other operations for thoracic diseases. So, their knowledge about male breast cancer did not update fast. And, our patient still performed radical mastectomy.

Neither adjuvant chemotherapy nor radiotherapy guidelines have been studied clearly in patients with ACC of the breast. Although no conclusions have been drawn, the 2011 St. Gallen International Expert Consensus voted that for node-negative breast ACC, adjuvant chemotherapy is not recommended. Radiotherapy is administered when breast-conserving treatment is undertaken, or a large tumor with affected lymph nodes is present [11]. Neither the previous eight reported cases of breast ACC in males nor our present case undertook adjuvant chemotherapy or radiotherapy.

Hormonal treatment and antiHER2 treatment are rarely used, because hormone receptors and HER2/neu expression are usually absent. In the view of the extreme rarity of male ACC of the breast, it is difficult for clinical trials. The standard treatment for male ACC of the breast is only based on those for female breast ACC. In the present case, tamoxifen was administered following the operation because the estrogen receptor was 3% positive in the biopsy. But he stopped taking tamoxifen after 13 months because of sexual dysfunction. Since it is a rare neoplasm, the optimal treatment and the value of adjuvant therapy need to be further investigated.

Generally, female breast ACC has an excellent prognosis compared with other forms of breast cancer and ACC of the salivary glands [24]. Female breast ACC does not show the aggressive characteristics of adenoid cystic carcinoma of the salivary gland and has a low local recurrence, a low axillary lymph node involvement, and a rare distant metastasis [11]. The 10-year survival rate for patients with ACC of the breast in females has ranged from 85% to 100% [2].

There is very little information is available on the outcome of this tumor in the male breast ACC. However, the prognosis of male breast ACC may not be as good as females.

In the published cases, the follow-up period of most breast ACC cases in males was relatively short. Our present case is well 67 months after radical mastectomy. Of the eight cases [3-10] in Table 1, two patients had recurrence. And, two patients had developed multi-organ distant metastasis. The follow-up period of two cases was not given clearly. According to all this data, male breast ACC seems more aggressive than female breast ACC. The prognosis of male breast ACC should be reconsidered, and the treatment might be strengthened.

**Table 1:** **Summary of eight reported cases of breast adenoid cystic carcinoma in males**

**Author**	**Publication year**	**Age (yr)**		**Side**		**Axillary lymph node**		**Treatment**		**Outcome of follow-up**			
Woyke [7]	1970	37		?		Neg		Local excision (ND)		Recurrence at 5 and 7 year			
Verani [8]	1973	78		R		Neg		Modified radical mastectomy (AD)		DOD at 9-month lung metastase			
Ferlito [9]	1974	60		L		Neg		Simple mastectomy (ND)		Not available			
Hjorth [10]	1977	21		L		Neg		Simple mastectomy (ND)		A & W at 2 years			
Miliauskas JR [5]	1991	13		R		Neg		Subcutaneous mastectomy (ND)		A & W at 30 months			
Kshirsagar AY [3]	2006	82		L		3/5		Modified radical mastectomy (AD)		Recurrence at 2 years			
Liu J [4]	2012	20		R		0/3		Simple mastectomy (AD)		Not available			
Yoo SJ [6]	2013	41		L		Pos		Not available		Bone and lung metastasis			
The present case	-	19		R		0/41		Radical mastectomy (AD) tamoxifen		A & W at 67 months			
Summary	-	10–29 years	4	L	4	Pos	2	Surgery	8	LR		DM	
		30–49 years	2	R	4			RT	0	Yes	2	Yes	2
		50–70 years	1	?	1	Neg	7	CT	0	No	5	No	5
		70–90 years	2					HT	1	?	2	?	2

*ND* no axillary dissection, *AD* with axillary dissection, *A & W* alive and well, *RT* radiotherapy, *CT* chemotherapy, *HT* hormone therapy, *LR* recurrence, *DM* distant metastasis, “*?*” unknown, “*pos*” positive axillary lymph node, “*neg*” negative axillary lymph node.

## Conclusion

As breast ACC is a very rare tumor, especially in males, we believe that this report will add information regarding its knowledge and management.

## Consent

Written informed consent was obtained from the patient for the publication of this case report and accompanying images. A copy of the written consent is available for review by the Editor-in-Chief of this journal.

## Abbreviations

ACC: Adenoid cystic carcinoma
RM: Radical mastectomy
PR: Estrogen receptor
ER: Progesterone receptor
USG: Ultrasonography
HER2: Human epidermal growth factor receptor 2
IHC: Immunohistochemistry
